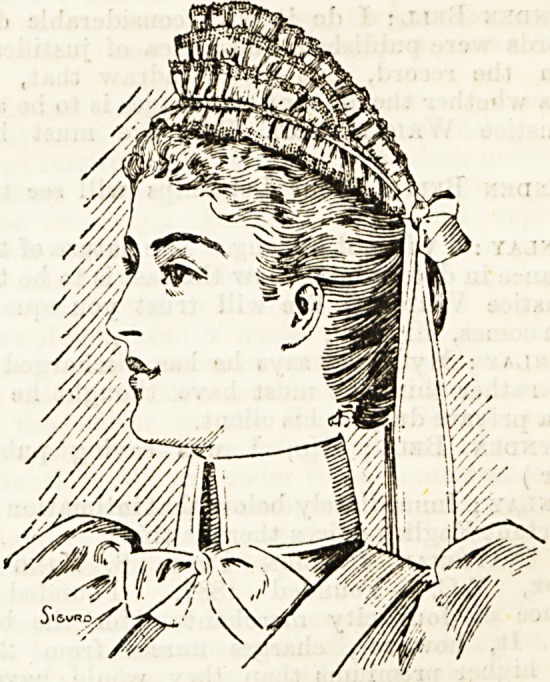# The Hospital Nursing Supplement

**Published:** 1894-04-14

**Authors:** 


"The Hospital" Nursing Mirror.

Being the Extra Nursing Supplement of "The Hospital" Newspaper.

[Contributions for this Supplement should be addressed to the Editor, The Hospital, 428, Strand, London, W.C., and should have the word "Nursing" plainly written in left-hand top corner of the envelope.]

### News from the Nursing World.

### WHERE TO SAVE.

When anyone's income, be it a large or small one, undergoes a reduction, retrenchment in some form or another is naturally contemplated. Perhaps in no way do individual weaknesses exhibit themselves more strongly than in such crises as these. "Well, father," said a young dandy to his parent last year when times were particularly bad, "if you've lost all this money, you'll have to cut down your charities!" "Nay," rejoined the old man soberly, "Not our *charities*, son, only our *luxuries*."

### USEFUL LIVES.

For twenty years Miss Macpherson superintended the work of the Belfast Society for Nursing the Sick Poor in their own homes. She was chiefly instrumental in organising the society, which has steadily increased and prospered, thanks to her energy and that of the ladies and gentlemen associated with her in the work. Only a few months back her health obliged her to resign the active superintendence, and arrangements were therefore made for its being carried on in a satisfactory manner. The work begun in Belfast has been copied in other parts of Ulster, and the Home for the District Nurses, of which the opening was duly noted in our columns, was truly a fitting memorial of Miss Macpherson's labours. Her death on March 28th of pneumonia caused deep grief to an immense circle of friends. Another recent death in the nursing ranks in Ireland was that of Miss Isabella Wynne, night superintendent at the South Infirmary, Cork, which took place after a few days' illness. She had been connected with the staff of the infirmary for two and a-half years, and her funeral was attended by the whole medical staff as well as by the nurses and other friends to whom she had greatly endeared herself.

### OLD LINEN.

Old linen is just as much wanted as ever it was, and there appears to be far less of it available now-a-days. Obviously in these hygienic times woollen materials have largely superseded the fine Irish linen in which our grandmothers, aye, and our own mothers, too, took such pardonable pride. If the use of calico, as well as linen, has greatly decreased, so that people have to own regretfully that they are unable to comply with the constant pleas of the district nurses for fresh supplies of clean rags, then assuredly a substitute must be found for the nearly obsolete material. An equivalent is urgently demanded if the nurses' work is to be properly done and the patients satisfactorily tended, and it is quite easy for the kindly disposed to temper unwilling negatives with donations which will enable the sorely pressed district nurse to purchase such stores as will compensate her patients for the unattainable "old linen" of former years!

### A SUCCESSFUL BAZAAR.

The Victoria Hospital at Folkestone was the scene of a brilliant gathering on the last Wednesday in March, when a bazaar, organised by Mrs. Percy Lewis, passed off most successfully, and by the funds raised on the occasion, the debt on the hospital was removed. Lady Katharine Eustace opened the bazaar, and Mrs. Percy Lewis was assisted by a large band of excellent helpers, who may well congratulate themselves on the results of their labours.

### WHERE WAS THE DOCTOR?

According to the local press, the Coroner of Runcorn has seen fit to dispense with an inquest in the case of a child who died after a brief illness, because of a remark made by a district nurse. She had seen the two months' old baby "some time previously, informed the police that he had a delicate constitution, and she concurred with the opinion that convulsions were the cause of death. The Coroner, under these circumstances, did not deem an inquest necessary. . . . He should have held an inquiry but for the testimony of the nurse." Is there no doctor in Runcorn to give death certificates? However cordially we may agree with the Coroner in his opinion of a district nurse being "a most valuable and useful institution," we cannot think it fitting that responsibilities so distinctly outside her own province should be thus laid upon her.

### GLOUCESTER.

It was announced at the annual meeting of the Gloucester District Nursing Society that the Board of Guardians had proved their satisfaction with the work done amongst the sick poor by the nurses for they had doubled their subscription to the society. A Home for the nurses is urgently required, and will doubtless ere long be secured for them.

### A WISE STEP.

The Local Government Board is powerfully advocating the efficient nursing of the sick poor in all parts of the country. The employment of outdoor nurses by Boards of Guardians is surely a particularly useful scheme, and we find that the Local Government Board is authorising the appointment of these special district nurses. The scheme is certainly humane and economical, and should be widely adopted.

### PROPOSED TRAINING AT CHELMSFORD.

The advisability of forming a County Nursing Association in Essex was discussed at a meeting at Chelmsford Shire Hall on March 30th. Dr. Thresh and Miss Lucy Chancellor suggesting the establishment, in connection with the infirmary, of a school of instruction in practical nursing and hygiene. Lord Rayleigh was in the chair, and Lady Rayleigh was amongst those present. The scheme, as proposed by Dr. Thresh, appeared to embrace the selection of suitable women to undergo a period of three or six months' training of combined ward and district nursing, supplemented by public lectures.

Such instruction would be of enormous value to the pupils, rendering them most useful members of the community, but it is obviously far too limited training to justify the assumption of the name of nurse. If it is thought essential that cottagers should be nursed by attendants of their own class, these should certainly receive an equal, not a shorter period of training than that considered necessary for the intelligent and educated women, who have already taken up this branch of work with marked success. Doubtless this point will receive the consideration which its importance demands from the Chelmsford committee, which it is proposed to compose of a hundred ladies and gentlemen, "and any others who wished to join," as Dr. Thresh is reported to have said at the preliminary meeting.

### SECTARIAN CHARITIES.

At a meeting held last month in Dublin in connection with the proposed reopening of the Holles Street National Lying-in Hospital, subscriptions to the amount of £400 were announced. Archbishop Walsh promised £100 a-year for ten years if the hospital were established on the larger scale proposed. The Lord Mayor of Dublin accompanied his subscription of £5 with frank regrets that the governors of the Rotunda Hospital should by practically excluding Catholics from any share in the management of that useful institution have obliged them to contemplate the establishment of a rival hospital.

### CONVALESCENTS AT TEESIDE.

The Stockton and Thornaby District Nursing Association have been considering a proposal to purchase Pemberton House, with four acres of ground, as a convalescent home for Teeside, and it has been decided to rent the house furnished for six months with the option of purchase. The Association is to be congratulated on an acquisition which cannot fail to largely increase its usefulness.

### THE CAMA HOSPITAL.

The hospital proper at Cama, with the neighbouring building for obstetric cases, holds about 100 patients including children, for whom a special ward is reserved. It is looked upon as a particularly good nursing school, and about sixteen probationers are in course of training, nine others having obtained certificates of efficiency last year. At first twelve months was considered as long a period as pupils would be willing to give to training, but now a year and a half is the minimum fixed on, and just as many applications are received, which certainly proves that the idea of prolonged training is not unacceptable to the natives. Of the eighty women who have passed through the school since it was first established, only about six have since abandoned the nursing profession. It is particularly satisfactory to find that the native women are now generally maintained during their training by their own relations, instead of its being done, as at first, by English funds. It is not only as economy to the hospital that this fact is welcomed, but also as evidence of the increasing value attached by the population to systematic instruction in the art of skilled nursing.

### AN APPEAL FROM INDIA.

Two qualified lady doctors have the management of the Victoria Hospital, Benares, where native women and children are treated both as in and out patients. In the latter department the weekly average of those who receive advice and medicine free is two hundred and twenty-three. The people, by steady degrees, have grown to put great confidence in the aid which was first placed at their service in 1890. Twenty-two cots are endowed and they are constantly full; in fact, sick women sometimes plead pitifully to be taken in, and when they learn that there are no vacancies, they beg for leave to lie out in the grounds until one occurs. There are still some unsupported beds, or "cots" as they are ordinarily called at Benares, and these have of necessity been requisitioned during the past year. This has caused a great drain on the reserve fund, which will, it is estimated, be entirely exhausted by the middle of the present year unless the appeal of the managers of the Victoria Hospital is responded to promptly. In addition to maintenance for the existing "cots," increased accommodation is also sorely needed. Ten pounds a year suffices to support a cot. Therefore ten annual subscribers of this very moderate sum provide for not ten patients only, but a constant succession of sick and suffering women in the ten cots during each year.

### TRICYCLES FOR DISTRICT NURSES.

The use of a tricycle has been adopted by certain district nurses in a part of the country where long distances have to be traversed. They say that they find it of the greatest possible use in economising their time and strength. A light receptacle under the seat carries the "district bag" most conveniently. During several months of the year the state of the roads precludes the use of the tricycle, but possibly one of the residents in the neighbourhood will see his way to making the nurses a loan of some other vehicle for that period. Women so bent on helping themselves certainly merit a little friendly assistance.

### A MUSICAL TREAT.

On Sunday afternoon an organ recital took place at the Queen's Hall, Langham Place, at which some two thousand people had the pleasure of listening to selections from the works of Handel, Mendelssohn, Beethoven, &c. The audience enjoyed with the deepest and most reverential attention every item on the excellent programme provided for them. The rumour that the free organ recitals at the Albert Hall were to be suspended appears to be without foundation.

### SHORT ITEMS.

Mrs. S. J. Smith has been appointed Head Nurse of the Grimsby Workhouse. There were two applicants for the post.—A concert held on the 2nd inst., in aid of the funds of the Radstock Sick Nursing Society was very well attended; it was got up by Mr. Joseph Deely.—At Penrhyn Hall, Bangor, a most successful bazaar, under distinguished patronage, has been held on behalf of the Nursing Institution.—At the annual meeting of the Diocesan Institute for Trained Nurses at Salisbury, it was decided that "diocesan" should be omitted from the title in future, as it gave an erroneous impression as to the extent of the work, which now covers a very extensive field.—An excellent address was given by Lady Victoria Lambton at Haverford West the other day, in which the necessity for fully trained district nurses in rural districts was admirably detailed.

### On General Nursing.

By Rowland Humphreys, M.R.C.S., L.R.C.P.Lond.

### VII.—CATARRH (*continued*).

### Dangers.

The principal dangers connected with a catarrh attacking the nose lies in its extension to the neighbouring parts. To infants there is a real danger in the nasal affection, in that the child is unable to take bottle or breast, and its respiration, as in the lower animals, is almost confined to that carried on through the nostrils. The author has occasionally experienced considerable anxiety for his little patient under these circumstances. The amount of dyspnœa resulting from the impediment in the nose causes great distress, and the baby has to be taught to breathe through its mouth. Still coryza does not very frequently attack them.

In older children we have to be on the look out for obstruction to the glottis, or chink between the vocal chords through which the air passes in breathing, from the swelling of the mucous membrane of the larynx, which may cause very rapid death. Laryngeal catarrh may also set up spasm of the glottis, the child being choked, before help in the form of tracheotomy can be obtained; or the complaint may extend to the bronchial tubes, and set up bronchitis, or extend still further to the alveoli, the actual working parts of the lung in respect to its special functions (*i.e.*, the elimination of the waste gases, the oxidisation of noxious matters in the blood, and the aëration of its oxygen-carrying material).

The laryngeal affections are known by their causing a change in the voice; it becomes hoarse, or muffled, or brassy, and in bad cases the respiration becomes markedly prolonged, and there is evidently great difficulty experienced in breathing, and symptoms of heart failure, as shown by irregularity in the pulse, blueness of face, nails, &c., inability to lie down, and great restlessness, call urgently for some operative interference. The bronchial symptoms are similar, except that there is little or no alteration in the voice, while there is wheezing on the chest, which can be readily heard by making the child open its mouth wide and breathe deeply whilst the ear is applied to the chest either in front or at the back. The symptoms, if severe, point to bronchitis, if mild and unaccompanied by marked rise of temperature or respiration, and if there is no movement of the nostrils in breathing, rather to the occurrence of bronchial catarrh than of broncho-pneumonia; that is to an affection of the surface of the bronchi rather than to the implication of their deeper parts. It is not so dangerous as the more severe complication. The onset of broncho-pneumonia is known by the considerable rise in pulse and temperature and the excessive and disproportionate rise in the respirations, the latter being also accompanied by marked movement of the nostrils. The lung becomes solid in parts, and on percussing or tapping the chest wall the note given out is of a dull, wooden character wherever this is the case. The patches of dulness are usually small in extent, but widely distributed, thus differing from acute pneumonia where a large continuous area of the lung is implicated. The material which blocks up the air vesicles is, in the case of broncho-pneumonia, catarrhal in nature; that is, it consists almost entirely of the epithelial cells and mucus, whilst in the acute form it is composed of inflammatory fibrinous exudation from the vessels in the walls of the air cells—smaller bronchi. Still, in children especially, it is a common thing to find some amount of dulness remaining for a long period after apparent recovery, and there are other indications that broncho-pneumonia, or catarrhal pneumonia as it is often called, implicates the substance of the lung as well as fills up its cavities.

The best way to prevent these complications is to take care of a cold in its early stages, and even if it does not definitely oblige the patient to lay up, yet it often leaves a catarrh of one apex of the lung which may pass on to phthisis or may leave a chronic cold of head or stomach which paves the way to other disorders by keeping the patient weak.

In the acute stage of a coryza the afflicted person had best keep in bed, and may be made much more comfortable by putting a steam kettle on, giving plenty of liquid or soft food and warm mucilaginous drinks. The bowels should be opened, and it is an advantage to douche out the nostrils with a hot solution of borax or some other antiseptic, or bicarbonate of soda, or salt. Ferrier's snuff, containing morphia, bismuth, &c., may be sniffed up into the nostrils, or the nose may be painted out with a two or four per cent. solution of cocaine. It is also said that the passage of a galvanic current through the cheeks relieves greatly. A hot foot-bath, with some mustard added, and a dose of 10 grains of Dover's powder, followed later on by some sweet spirits of nitre, solution of acetate of ammonia, or other similar drug, will hasten the stage of watery discharge, and bring the relief it affords. After the immediate attack is over there is often a loss of appetite and inability for work, which has to be combated by tonics and change of air. If the catarrh should descend to the stomach or intestines, a mustard plaster or linseed poultice, or a layer of cotton wool and a bandage is very comforting.

Acute catarrh of any of the mucous membrane may be caused by the presence of irritating material, and the stomach is not uncommonly affected in this manner by poisonous food, such as mussels under certain circumstances. The obvious treatment in these cases is to remove the irritating material substance by an emetic or douche, and then to apply some local sedative. Acute catarrh may pass off entirely or may lead on to the chronic condition affecting any of the organs or parts attacked or liable to it.

### Chronic Catarrh.

This is a very different, and in its results far more serious complaint than the acute form. In the first place it will be as well to describe the changes produced in a mucous membrane by a long-standing cold. The results vary in that they may be in the direction of an increase or of a decrease in the affected tissues, though the two are often found together.

In the stomach, for example, the mucous membrane in old-standing cases is thickened and pale, and shows signs of old ulceration, and of small hæmorrhages into its substance. Sometimes a general wasting is seen, sometimes there is an overgrowth of fibrous tissue, such as is seen in scars, and which contracts after a time, causing wasting of the parts enclosed in or pressed on by it. Ulceration occurs both in chronic and acute cases, but especially in the latter.

The glandular structures pressed on by the fibrous tissue, as already mentioned, waste; the surface of the mucous membrane affected is covered with a thick coat of mucus, which in the nostrils forms a hard crust; in the stomach it remains more or less attached to the surface, and causes the food to ferment rather than to digest, while it tends to prevent the action of the gastric juice, and so impedes digestion. In the intestines it presents the same appearance as the stomach, and not only prevents the proper onward movement of the food by making the wall too slippery, and thus preventing the onward movement of the food masses by the action of the muscles in its walls, but what is still more important, it prevents the absorption of the food by the special apparatus provided for that purpose, so that a person may be taking plenty of nourishment by the mouth, and yet be in a state bordering on starvation from the food not reaching the tissues. In the bronchi the results are as severe, and show more definite signs. As soon as the muscular lining to these tubes is affected and has wasted, the tube itself begins to dilate, and the greater the area of muscle affected, the greater is the dilatation produced. Here the mucus, which as elsewhere is far greater in amount than it should be, collects, and tends to decompose, the two conditions found in bronchiectasis. A similar condition in the nose is called ozæna. With this condition of chronic catarrh there is great tendency to acute attacks, so that "colds in the head," attacks of bronchitis, of broncho-pneumonia, gastritis, and acute intestinal catarrh constantly recur. Alcoholic people are especially liable to those recurrent catarrhs, and they frequently lead directly to the actual cause of death.

In rickety children catarrh is a great source of danger. Rickets is usually due to some digestive defect combined with catarrh and caused by improper feeding, and the nourishment as well as the growth of the child is thereby affected. All the chronic catarrhs are rendered much more severe and troublesome to cure by the presence of constitutional disease, especially syphilis and scrofula, and in the lung a catarrh, by preventing the proper entrance of air, causes slow collapse of that part of the lung whose tube is blocked, and renders it an excellent spot for the development of the ubiquitous tubercle bacillus.

In the pharynx the presence of a chronic catarrh usually causes some degree of irregular overgrowth, and affects the voice, causing a permanent hoarseness, or hoarseness may only come on after or while using the voice; improper use of that organ, as is so commonly seen in clergymen, constant "nipping," and such like are the usual causes of such troubles.

Other causes of chronic catarrh are excess in eating, irregular meals, too great luxury, debilitating circumstances of any description, and such like.

The treatment in chronic catarrh resolves itself into antiseptic and astringent local applications, the avoidance of the particular cause, and, where circumstances permit, plenty of good food, change of air, avoidance of worry (often at the bottom of an old-standing catarrh), rectification of any hereditary or acquired condition which is causing nerve strain (as, for example, in the eyes), the keeping of the skin clothed all over (that is, up to the throat) in flannel or at least merino both in winter and summer, regular exercise, &c.

Unfortunately the very persons who most require to take care of themselves are often those who are least able to do so either from the nature of their occupation or from some defect in their powers of self-control. For the latter there is nothing like putting the patient under the care of some properly trained capable nurse, who will educate him to take care of himself, without producing a hypochondriac.

### A Nursing Home at Kew.

"But private Nursing Homes are so expensive," is the objection constantly offered by patients of moderate means when the doctor recommends their going into one. This is unfortunately often quite true, and therefore it is well to go and see any establishment which offers comfortable care and clean and pleasant surroundings for sick people of limited incomes. There is a home of this kind at Kew, not far from the station, and also near to the beautiful and well-known gardens. It is kept by Miss Chapman, a nurse who herself looks after the patients. The terms are extremely moderate, and can be obtained by application to Lawn View, Lawn Crescent, Sandycombe Road, Kew.

### The Ethics of Nursing.

By Louis Vintras, M.B., B.Sc., Licentiate of the Royal College of Physicians, Member of the Royal College of Surgeons, Resident Medical Officer to the French Hospital.

I.

Medicine has justly laid claim to the double title of being a science and an art; the same may be said of nursing, it is a science because it requires precision, it is an art because it demands a vocation. A nurse who merely looks on her calling as an occupation can never hope to perform her duties in a way satisfactory to the patient or creditable to herself. Her life must be one of careful observation, enlightened by a deep sense of charity, with the word "Abnegation" for its motto.

The duties of nursing are not such as can be undertaken by all; they require knowledge based on an intelligent conception of the varied items of the work, and this can only be attained by the expenditure of a certain amount of intellectual power. The days of mechanical nursing are passed, and we expect something more than mere routine from those who have striven to elevate nursing from what was little better than servitude to the dignity of a profession. It is to the nurse that the medical man looks for the execution of a multitude of details that can only be done by a person who is constantly with the patient, and the importance of which cannot be overestimated. This for two reasons: First, the treatment of a case can only be thoroughly effectual on condition that it shall be carried out with a due respect to minutiæ; second, this constant care surrounding a patient has a salutary influence on his imagination, and the imagination of patients is a factor it would be almost impertinent to ignore in these days of psychical research.

"The keeper of the house of life is fear . . ."

has said Robert Louis Stevenson. This may be very true in health, fear may cause man to take certain precautions to guard himself against illness, but in the diseased state the house of life is more or less endangered already, fear becomes a depressing adjunct, and, medically speaking, we prefer confidence as our ally. It is this confidence that the nurse especially can do much to promote and also in some cases much to lessen. A patient is an inferior being, his mind for the time is weakened, and like all feeble mortals he is easily led by the least show of authority; but a nurse can have no authority with her patient unless she can show that she knows herself how to obey. By her implicit obedience she will heighten the confidence of the patient in his physician, and a part of that confidence will be reflected back on herself.

So the nurse must recognise in the medical man her scientific chief, and it is only by assuming this view of her position that she will thoroughly understand the importance of the duties she has undertaken, and comprehend the necessity of that rigid discipline that should not be second even to that of the soldier, for in the warfare with disease so on the battle-field, it must ever be remembered that it is the lives of human beings that are entailed.

A sense of duty, an absolute obedience to orders, a thorough comprehension of these orders, are the fundamental principles of nursing. I cannot insist too strongly on the last point; there is a tendency among beginners to think they have understood, and a kind of fear that their intellectual reputation will suffer by seeking information or by causing directions to be repeated. It is an error. A nurse will never be blamed because she does not understand; but she will be blamed later on because she has not understood.

The life of some women of fashion consists merely in an intimate acquaintance with the process of converting trivialities into State functions. I should not like to say anything against trivialities, they are very pleasant things, but the art of nursing knows them not. The smallest stone in the building has its utility, and is therefore important, so has the smallest detail in the treatment of patients.

### The Nursing of Diphtheritic Paralysis.

### THE AFTER-CARE OF PATIENTS.

By the Matron of a Children's Hospital.

So much has been written on the subject of diphtheria that there seems to be nothing further to be said on the subject; but on the after-care of diphtheria patients there is much yet to be learned, and it is a matter which requires careful thought.

In all cases of diphtheria, when the membrane has disappeared, and after tracheotomy, when the tube has been removed and the wound closed, we are apt to feel that we are relieved from all further anxiety on the patient's account. But we must not forget that the whole system has been lowered by a disease from which the patient is recovering, and we must not allow any undue exertion on the patient's part. More especially is this precaution necessary in the case of children, for it is in our children's hospitals that so many cases of diphtheritic paralysis are seen, such cases being sent in from the out-patient department.

### Symptoms and Treatment.

One of the first symptoms noticed by the parents is the change in the tone of the voice, the voice becoming nasal; then the child cannot walk properly, and falls about, and there may be difficulty in swallowing.

Our first care is to keep the patient as quiet as possible. This is not at all a difficult matter if the paralysis is in an advanced stage, for the child lies in a most listless state, apparently utterly indifferent to all that is going on around. Should, however, there be any trouble in keeping him quiet, this may be overcome by fastening the child down in bed by means of a broad band of webbing passed across the mattrass and tied with tapes to the bedstead. A narrow piece of webbing is then placed across the chest, to the ends of which two pieces of narrow webbing are fastened placed crosswise, these pieces going round the upper part of the arms and the webbing which is tied across the bed; this webbing is fastened round the arms by means of buckles. This will be found to most effectually prevent the child from sitting up, and it very soon gets accustomed to the constraint.

### Administration of Nourishment.

Our greatest difficulty is in giving nourishment, for there will probably be regurgitation of food, especially of liquids, which we must give most carefully, in very small quantities, and with a teaspoon, in case the fluid should get into the trachea and choke the patient. We shall probably find that solid food is better tolerated, and then it must be given in as nourishing a form as possible—fish, or chicken panada, boiled or baked custards, &c.

Should, however, the patient be unable to swallow without choking, we shall find it best (with the doctor's permission) to use the nasal tube, for we can by this means administer nourishment regularly and more satisfactorily than in any other way, and although the patient strongly objects to the process at first, we can always by coaxing and gentle persuasion render him amenable to our wishes, and it is wonderful how quickly children learn to submit to the treatment. The method of using the nasal tube is described in nursing books, but perhaps a few words may not be out of place here. A soft rubber catheter is used for this purpose, to the open end of which is attached a glass funnel. The catheter end, after being well coated with glycerine, is passed through the nose, down the back of the throat into the œsophagus; some irritation is caused as the tube passes down the back of the throat, and one's first impulse is to withdraw the tube, but it will be found wiser to continue its introduction, at the same time trying to quiet the patient. When this has been done the nourishment is gently poured into the funnel. If milk is given it should either be partially peptonised or mixed with lime water or barley water, and nourishment should in all cases be given lukewarm. The tube should be quickly withdrawn, and after having been syringed through with warm water should be left soaking in a weak boracic solution, that it may be ready for use when again required. Practice alone can render anyone skilful in the use of the nasal tube, but it will be found a most valuable aid in nursing.

### Important Points.

The most important points to be observed in nursing diphtheria after membrane has disappeared are:—

1. Change in the tone of voice.
2. Regurgitation of food.
3. Irregularity of pulse, which is often intermittent and should always be reported to the doctor.

The drugs and the amount of stimulant must be left to the physician, but good nursing is most important in all cases of this nature, and too much stress cannot be laid on the duty of enforcing absolute rest, and of most careful attention to the administration of nourishment.

### Novelties for Nurses.

### A PRETTY CAP.

(Messrs. J. and J. Cash.)

Cash's frilling is well known to all of us, and its usefulness and durability duly appreciated. Further, we know that Messrs. Cash are always producing novelties and keeping the varying wants of nurses, nurseries, and women in general in mind. We used to have to utilise our frilling in the matter of caps ourselves, and although the use of the frilling was a saving of time, yet to sew it on neatly is a labour we have not always leisure for. Messrs. Cash seem now determined to save us even this trouble, and caps can be procured ready made from their firm, made so neatly and securely that the most fastidious in the matter of strong and finished needlework should find nothing to complain of. We append an illustration of a charming cap Messrs. Cash are now supplying. For the waitress or children's nurse nothing could be prettier or more suitable; for nurses who regard the cap as a covering for the hair a little modification is necessary. We feel sure, however, that any hospital or institution sending an order for a number of caps could have their own design carried out, and the result would be a durable, dainty cap, securing uniformity not attainable when caps are left to by made by different individuals. We are convinced such a course would give satisfaction to any institution.

### Nurses' Friends and Foes.

THE ROYAL NATIONAL PENSION FUND FOR NURSES v. THE RECORD PRESS (LIMITED).

In the High Court of Justice, Queen's Bench Division, Royal Courts of Justice, Tuesday, 10th April, 1894. Before Mr. Justice Wright and Mr. Justice Bruce.

Mr. Finlay, Q.C., said: My Lords, I appear, with my friend Mr. Longstaffe, in support of an appeal from a decision of Mr. Justice Kennedy at Chambers. This action is one brought by the Royal National Pension Fund for Nurses, for whom my friend and myself appear, as plaintiffs, against the defendant company, called the Record Press (Limited). The plaintiff association is an association that was formed for the public purpose of providing a scheme of insurance for those engaged in the profession of nurses, to enable nurses to insure themselves against things incident to their profession. For that purpose a number of wealthy gentlemen in the City came forward with a very large subscription, provided a sum of about £50,000 to enable this association to be conducted to the utmost possible advantage of the nurses who were to be benefited by it. It is not a trading association. The defendant company is a limited company formed for the purpose of printing literature very largely connected with the profession of nursing. The libel which forms the subject of the action is one for which the defendants now do not pretend there is any justification. On the 22nd July the defendants published this libel in two publications. One publication is in a little book called, "How to Become a Hospital Nurse," and the other is in what they call the "Nursing Directory for 1893." In both these publications there appears this statement, which I will read without comment:

"Nursing Record" Fund.—Annuity and sick pay given by two important English offices at ordinary commercial rates. Application to the Editor, *Nursing Record*, 376, Strand, W.C.

That is an intimation that there are two important offices which may be communicated with through the Editor which will give insurance at ordinary commercial rates. Then, immediately below, there is this ——

Mr. Lynden Bell: I think I am performing a public duty in saying that this is wholly immaterial to any point which your Lordships have to decide.

Mr. Justice Wright: I think we must trust Mr. Finlay for that.

Mr. Lynden Bell: I do it with considerable deference. These words were published, and a plea of justification was put upon the record, but we withdraw that, and the question is whether the amount of damages is to be assessed.

Mr. Justice Wright: Mr. Bell, we must hear Mr. Finlay.

Mr. Lynden Bell: Your Lordships will see that I am right.

Mr. Finlay: I will not be long. The nature of the case is of importance in determining how the case is to be tried.

Mr. Justice Wright: We will trust you equally when your turn comes, Mr. Bell.

Mr. Finlay: My friend says he has discharged a public duty. I rather think he must have thought he was discharging a private duty to his client.

Mr. Lynden Bell: No, I was saving public time. (Laughter.)

Mr. Finlay: Immediately below that intimation about the two important English offices there is this:

Royal National Pension Fund, 8, King Street, Cheapside, E.C. — Founded 1887. Founded by the munificence of four city merchants "for the benefit of nurses." It, however, charges nurses from 20 to 26 per cent. higher premiums than they would have to pay for similar advantages at old-established London offices, which, under the circumstances, and the many assurances that this is a benevolent scheme, is somewhat surprising.

Of course one object of that is that all nurses should be induced to communicate through the Editor with the old-established insurance offices, which were prepared to insure at rates which the Editor said were 20 to 26 per cent. lower than those charged by this benevolent institution. In respect of that statement a communication was made to the defendants, and this action was brought. On the 23rd December, 1893, the defendants delivered their defence, denying that they published the words "falsely or maliciously," or that the words bore the alleged meaning or any of them; and in the alternative the defendants justified, and said that the words complained of in their proper and ordinary meaning were true in substance and in fact.

Mr. Lynden Bell: That is amended.

Mr. Finlay: I am told there was an amended defence, but I think that must be in my friend's breast. It was never delivered. That was the only defence which was delivered. Then particulars were asked for of the institutions which were ready to insure at the rates mentioned, and the name given—the only name given in those particulars—was the North British and Mercantile Insurance Company. These particulars were delivered on the 20th February, 1894. On the 24th February, 1894, four days after the delivery of these particulars, the defendants took out a summons before Master Archibald and obtained from him this order. Your Lordships will find it, I think, bound up with the pleadings. "Upon the application of the defendants, and upon hearing the solicitors for the applicants and for the plaintiffs, and upon reading the writ issued in this action on the 17th day of August, 1893, the statement of claim delivered on the 5th day of December, 1893, it is ordered that this action, commenced in the Chancery Division of the High Court of Justice, be transferred to the Queen's Bench Division of that Court, if the President of that Division shall consent thereto." That was followed by the order of the 28th February in these terms. It is an order made after the transfer to the Queen's Bench Division: "It is ordered that the defendants may be at liberty to withdraw their defence herein, and that the costs of and occasioned by the application be paid by defendants to plaintiffs." That order was made on the 28th February. It was drawn up and served by the defendants on the same day, but the defendants did not withdraw the defence. They took no steps at all, and on the 3rd March —three days afterwards—the plaintiffs gave notice of trial and set down the action for trial. On the 8th March the defendants took out a summons to set aside the notice of trial and to withdraw the record; and on the 12th March the Master made the order setting aside the notice of trial and ordering that the record should be withdrawn. That Order was affirmed by Mr. Justice Kennedy on the 15th March, and from that affirmation of the Order by the learned Judge, we come on appeal to your Lordships.

Mr. Justice Wright: Why should the record be withdrawn?

Mr. Finlay: Because we want the case disposed of. This is a libel which has been published for a purpose. We ask your Lorships to say that the ground upon which, as my friend says, the Master seems to have gone, to some extent at all events, that the order of the 28th February was equivalent to a withdrawal of the defence, was an entire mistake. All that the order of the 28th February did was to say this, "that the defendants be at liberty to withdraw their defence." Till the 12th March they did not act upon that. Upon the 12th March, 1894, they send this letter to the plaintiffs:

"Record Press (Limited).—The National Pension Fund, &c.—I beg to give you notice that, on behalf of my clients, I hereby and do withdraw the defence delivered herein on their behalf, in pursuance of the order made herein."

That was not until 12th March. In the meantime, the defendants not acting at all upon this leave which they had got on the 28th February, we delivered notice of trial, and I shall ask your Lordships to say that this is a case which ought not to be sent down to the Sheriff's Court to have damages assessed. This is a matter in which we shall ask your Lordhips —and it is for that reason I call attention to the nature of the action—to let the case be disposed of in the High Court under the power which your Lordships have, even if the defendants do now withdraw their defence.

Mr. Justice Wright: Is it your object to get an interlocutory injunction?

Mr. Finlay: We want an injunction.

Mr. Justice Wright: Is that the real substantial object?

Mr. Finlay: We also want this case tried in such a manner that nurses shall know the nature of this attempt that was made to deceive them.

Mr. Lynden Bell: We have offered all along, before the

Nurses' Friends and Foes—*continued.*

Master and before the Judge, to consent to the injunction, which, of course, is mere surplusage in this claim. The whole point is as to damages. We are quite willing to meet them before the Sheriff, where we say they only can go, and I am prepared to argue that. But, as to the injunction, we have all along, both before the Master and before the Judge, consented to the injunction, and Mr. Justice Kennedy, with his pen in his hand, was prepared to make it part of the order; but they said, "Oh, no."

Mr. Finlay: My friend is entirely mistaken in saying that all along they have offered to submit to an injunction. Before Mr. Justice Kennedy or before the Master they did say they would submit to an injunction. Your Lordships should know that this is not the only time this statement has been made, and made obviously for business reasons, by the defendants, because in the defendants' *Nursing Record* of the 10th August, 1893, there is an advertisement headed,

"Annuity Fund and Sick Pay Fund for Nurses. Figures for nurses to note and remember. For the same annuity nurses have to pay from 20 to 26 per cent. more to the Royal National Fund for Nurses than old-established and wealthy insurance offices demand." Then there is this: "Every nursing institution should send a stamped addressed envelope to the Editor, *Nursing Record*, 357, Strand, London."

I submit to your Lordships that this is not a case in which the defendants ought to be allowed to have it sent down to be tried at the Sheriff's Court. It is a case which should be disposed of in such a way that it will let the nurses know the attempts which have been made to mislead them in certain interests in regard to this matter of insurance.

Mr. Justice Wright: How can that be done, Mr. Finlay? How can we force the defendants to appear or to be represented in any way at the trial?

Mr. Finlay: I do not ask your Lordships to do that; but your Lordships have power to say that instead of sending the case down to the Sheriff's Court, where it will be tried before the Sheriff, the case shall be tried in the High Court.

Mr. Justice Wright: You are willing to do that at your expense, are you?

Mr. Finlay: The expense, of course, must fall upon the defendants if they are in the wrong.

Mr. Justice Wright: Why, if they admit they are in the wrong, should the expense of this fall upon them?

Mr. Finlay: The expense of properly trying the case is a legitimate expense. Your Lordships will remember there is power under Order xxvii., Rule 4. It was considered that it was in many cases not desirable that the cases should go down to the Sheriff's Court merely because the defendant was willing to withdraw his defence, and accordingly power was given in the concluding words of Rule 4, Order xxvii. in these terms: That the Court or the Judge may order that instead of a writ of inquiry, the value and amount of damages, or either of them, shall be ascertained in any way which the Court or a Judge may direct.

Mr. Justice Wright: But that is in order to ensure the proper estimation of damages, not in order to ensure publicity.

Mr. Finlay: Proper estimation of damages and proper trial of the case. Then, further, my Lord, we insist upon our right to an injunction.

Mr. Justice Wright: That they give you.

Mr. Finlay: Under these circumstances I submit that this order is certainly wrong. We are perfectly entitled to have this order set aside, because we were perfectly within our right in delivering notice of trial on March 3rd. The order of February 28th only gave leave to the defendants that they should be at liberty to withdraw their defence. Then it was not until March 12th that they sent this letter, which I will hand up to your Lordships, stating that they acted upon the leave which they had got. That is a letter saying, "In pursuance of the order we withdraw the statement referred to."

Mr. Lynden Bell: I do not know that I ought to say anything now, but it may save time. I know it is irregular, but it saves time. That letter was written after the proceedings before the Master by the solicitor's clerk. As these people before the Master pretended not to consider it withdrawn, he said: "Now I will let them know that it is." He writes that letter, but it is wholly immaterial, for this reason, that the order now appealed from is an order made by the Master and confirmed by the Judge, which order by the Master was made before that letter.

Mr. Finlay: Exactly, and that is what I am pointing out—that when we delivered notice of trial on March 3rd we were thoroughly justified in doing so, because all that the defendants had got was leave to withdraw the defence, and they had not acted upon that leave. Under these circumstances I submit it is impossible to sustain the order which was made in Chambers, and I would ask your Lordships if the defendants are now to be taken as having withdrawn the defence, which was not done, as my friend says, till after the summons was disposed of?

Mr. Lynden Bell: Yes, it was withdrawn, although the letter was not written.

Mr. Finlay: The letter was not written. But the order merely gives leave to the defendants to withdraw the defence. An order giving leave to the plaintiff to amend his pleadings, or to a defendant to amend his defence, does not, in fact, amend the proceedings.

Mr. Justice Wright: They would not have gone on after that. They were stayed.

Mr. Finlay: I am not sure.

Mr. Justice Wright: "Upon hearing the solicitors on both sides the Court ordered"——

Mr. Finlay: "May be at liberty to withdraw their defence herein." Until they acted upon that I submit that they might have delivered another defence. The plaintiff institution is not a trading institution, and it is not a matter of pecuniary loss or commercial interest to the plaintiffs. They are proceeding in this matter solely in the interests of the nursing profession.

Mr. Justice Wright: Yes; but this is simply wrongly worded. If you follow the wording of Order XXVI., Rule 1, it ought to have been worded: "It is ordered that the defendants do withdraw their defence herein." Under the rule the order should be that it be withdrawn. You cannot withdraw it without an order. It is simply a mistake in the wording.

Mr. Finlay: But at the same time, having got the order giving leave to withdraw, and not yet having done it, we could do nothing but give notice.

Mr. Justice Bruce: To go to what seems to be the substance of the matter: Mr. Finlay, do you contend that the damages could not be satisfactorily assessed before the Sheriff?

Mr. Finlay: I submit not, because points of some nicety may arise. We are, I tell your Lordships at once, a benevolent institution; we are not a trading institution, and no pecuniary damage is sustained by the plaintiffs in the sense of losing business. But we represent the nurses who were to be benefited by this fund, which was established by a number of the leading gentlemen in the City for the benefit of nurses, and we say that if a statement of this kind is made, which is absolutely untrue, and made for the purpose of leading nurses instead of insuring with this institution established for their benefit, to go, through the editor of the *Nursing Record*, to insurance companies, the nurses are being deprived of the benefits of this institution. We say under these circumstances, although we have not lost money as a commercial company would have lost money, we should be entitled to substantial damages as representing the interests to promote which this institution was formed.

Mr. Justice Bruce: What you really want Mr. Finlay, I take it, is to preserve the credit of the institution.

Mr. Finlay: Yes, that is all we want. But we do say that if an attempt of this kind is made, and made obviously for pecuniary reasons on the part of the defendant, to divert the nurses from an institution of this kind established for their benefit, we are not to be put off by being told, as we shall be told if we go to the Sheriff's Court, "You are not a trading institution; you have not sustained anything in the way of loss of dividends, and so on, and therefore you are only entitled to have nominal damages." I submit that we are entitled to have the ruling of the Judge upon a point of that kind. No doubt that is why the defendants are anxious to go to the Sheriff's Court. They mean there to take the point. They think that the trial at the Sheriff's Court, in the first place, is less public, and, in the second place, they desire to take the point that there is no loss of dividend,

Nurses' Friends and Foes—*continued.*

therefore, damages one farthing. That is the point that I submit, and I submit that is not a point to be disposed of by the learned Under-Sheriff.

Mr. Justice Bruce: If you get the character of your institution established, the actual damages would become immaterial. All you want is to have the character of your institution established.

Mr. Finlay: With this institution damages are nothing. All they want is to let the nurses be warned against these attempts which have been made to mislead them.

Mr. Justice Bruce: Could you not arrange now without the expense of a trial? The defendants, I understand, say they have no defence. The question as to the actual amount of damages you may be entitled to recover is probably somewhat difficult, but your object is not to recover a large pecuniary sum, but to establish the character of your institution.

Mr. Finlay: Certainly. I make again the offer which we have made already, and which the defendants have refused. If the defendants will make a proper apology, to be sufficiently published, and pay the costs of these proceedings, the proceedings will be terminated. We have offered that already, and if they refuse that I must ask your Lordships to allow this case to be tried in a proper way.

Mr. Justice Bruce: Published in the nursing papers.

Mr. Finlay: In the *Times* and in the nursing papers, and in Indian and Colonial papers, because it is specially in Indian and Colonial papers that this was intended to operate. There is a large amount of business and a large amount of commission coming from the colonies and from India, and this statement of the defendants was intended to operate upon that. All that we shall ask is that it should be published in such a form as to counteract the mischief that the original publication was intended to do.

Mr. Justice Wright: We have power, I think, to attach any terms to the leave to withdraw the defence. Any condition that the Judge ought to impose can be imposed here, I suppose, or by the Court of Appeal.

Mr. Justice Bruce: I do not quite know what "Indian and Colonial papers" means. Does it mean one or two leading papers in India? If it means an advertisement in all the papers published in India and the British Colonies it means a very heavy tax.

Mr. Finlay: I do not mean that.

Mr. Lynden Bell: Without in the least allowing Mr. Finlay's argument to be correct, or in any way giving up our resistance to this appeal, which I would ask your Lordships to dismiss with costs, I am perfectly prepared to do this with regard to this last suggestion of Mr. Finlay's. I am instructed to say that we admit we were in error. It was a quite *bonâ fide* mistake, but we were in error in our calculations, and we are perfectly willing to consent to judgment, together with an injunction, and to pay the costs of the action—not of these summonses and proceedings, but of the action—and also to pay costs of an advertisement to that effect in the *Times* and one other paper; but as to India and the Colonies in the present day, when the Empire is so very great, I think it would be a very severe thing to ask us to pay for advertisements all the world over.

Mr. Finlay: I am sorry to say that I cannot accept my friend's statement that it was a *bonâ fide* mistake. If the case was tried it would appear that it was nothing of the kind; it was inserted with knowledge and for a purpose. But as regards the advertisement I will say this. I mentioned Colonial papers because it was suggested to me. We are prepared to stand by the offer that we made in our letter of November 24th, which, I see, does not mention Colonial papers. It says that we offer to discontinue on their paying the costs, if they will withdraw the statement complained of, by an advertisement, the terms to be settled, such advertisement to be inserted twice in the *Times*, *Lancet*, *British Medical Journal*, Hospital, and the *Nursing Record.* I do not think there can be any objection to that.

Mr. Justice Wright: I suppose there is no objection to the plaintiffs advertising at their own expense in any papers they like.

Mr. Lynden Bell: Exactly. We were willing to advertise in the *Times* and one technical journal.

Mr. Justice Wright: It is to be distinctly understood the plaintiffs are not to run any risk by advertising the apology wherever they like, and as much as they like.

Mr. Lynden Bell: They must only state what is the fact.

Mr. Justice Wright: Whatever your apology is, they must be at liberty to use it as much as they please.

Mr. Lynden Bell: Certainly, my Lord.

Mr. Finlay: Perhaps your Lordship will allow this to stand over that the terms of the apology may be settled, and let it be mentioned again to your Lordship.

Mr. Justice Wright: What do you say, Mr. Bell? There is nothing to be gained by continuing the dispute.

Mr. Lynden Bell: I hope your Lordships will dismiss the appeal with costs. I was going to say I would insist, but I must press that I am prepared to argue this point, and to meet Mr. Finlay's argument on every point that he has laid before the Court. I submit that this order is perfectly correct, and that a fair trial could be had before the Sheriff and a jury. But since there is this short cut to a settlement of the action, we are willing to pay the costs of the action, and also to pay the costs of the insertion of the advertisement; but we are perfectly right in the course we have taken with regard to these summonses in Chambers and before the Judge, and I submit we are right here; therefore I will ask for the costs of this appeal.

Mr. Finlay: I can hardly consent to that.

Mr. Justice Bruce: It will be no advantage to you to go to trial, Mr. Bell, will it? because there certainly will be a verdict against you for something at the trial.

Mr. Lynden Bell: What I would advise him to do—I have not his consent—would be to leave the costs of the summonses below as they are, and say, No costs of this appeal. I would advise him to do that. But I may say I am rather under the strong impression that I have a complete answer. I do not want to weary the Court with an argument, but I think I can show.

Mr. Justice Wright: It sounds reasonable.

Mr. Finlay: Having regard to the motives which have led the Royal National Pension Fund to bring the case into Court, it is not worth while preventing what seems likely to be a satisfactory settlement of the action by a squabble about costs. Therefore, perhaps your Lordship will allow the matter to stand over, in order that the apology may be settled, and perhaps your Lordship, or one of your Lordships, will allow us to come before you to have the terms settled, in case there is any difference between the parties.

After further argument, it was ordered that the Record Press (Limited) should pay 40s. damages and the costs of the action, but not the costs of the appeal; costs below as ordered; an apology to be published in the *Times* and *Lancet* at the expense of the Record Press; the Pension Fund to be at liberty to publish the apology in any other paper at their own expense; the terms of the apology to be settled between the parties, but if they fail to agree to be settled by the Judge; and finally, an injunction was granted restraining the Record Press from any future publication of the libel.

Mr. Finlay, Q.C., and Mr. Longstaffe were for the plaintiffs, and Mr. Lynden Bell for the defendants.

### Minor Appointments.

Sheffield Union Infirmary.—Mrs. Amelia Chapman Lawson has been made Charge Nurse of the Maternity Block at this infirmary. She was trained at St. Mary's Hospital, Manchester, where she was afterwards Staff Nurse and Sister. She takes with her the good wishes of those with whom she has worked for a period of nine and a half years.

Wellington and District Cottage Hospital.—Miss Edith C. Mellor has been appointed Nurse-Matron of this cottage hospital. She was trained at St. Bartholomew's Hospital, London, and the General Hospital, Bristol, and we wish her success in her new work.

English Hospital, Rosario.—Miss Mercy Robinson has been appointed Nurse-Matron at this hospital.

Stranraer Cottage Hospital.—Miss Sarah E. K. Staveley has been made Nurse-Matron at this hospital. She was trained at the Royal Hospital, Belfast, and has our sincere good wishes.

### The North-Eastern Hospital for Children.

### DRAWING-ROOM MEETING.

A drawing room meeting on behalf of the North-Eastern Hospital for Children was held on the 11th inst. at Dr. Sansom's house in Harley Street. The spacious rooms were quite filled by an audience evidently deeply interested in the literary and musical entertainment provided. Dr. Sansom gave an account of the origin and growth of the hospital with which he has been connected for so many years, and for which he has done an amount of work the value of which it would be difficult to over-estimate. Some charming songs were sung by Mrs. Dyer and Miss Trask, and Canon Barker made an eloquent appeal in aid of this valuable and needy charity. Perhaps the special features of the afternoon were the readings given by Mr. Rider Haggard and Mr. Jerome K. Jerome. The former chose a chapter from "Jess," the magnificent scene in the bed of the river, which he gave with such spirit, and no one in the audience could agree in the author's suggestion that his first public appearance would probably be his last. On the contrary the exceedingly well-chosen words with which he commenced, proved him already a successful orator. Mr. Jerome treated his hearers to one of those happily chosen subjects which he has made his own. Under the title of "A Charming Woman," he described a type of a class with which we are, most of us, familiar. Like many of Mr. Jerome's sketches, it was absolutely true to life, and delicately drawn, and pervaded with keen satire. Mr. Jerome speaks on a platform as he does elsewhere, without any affectations or mannerisms, and his "reading" received most sympathetic attention from the first word to the last. The North-Eastern Children's Hospital in Hackney Road ought to benefit largely by the meeting so able organised by Dr. and Mrs. Sansom. We should advise our readers to lose no time in following the suggestion of Dr. Sansom to go and see for themselves the work done by this valuable institution.

### Notes and Queries.

The contents of the Editor's Letter-box have now reached such unwieldy proportions that it has become necessary to establish a hard and fast rule regarding Answers to Correspondents. In future, all questions requiring replies will continue to be answered in this column without any fee. If an answer is required by letter, a fee of half-a-crown must be enclosed with the note containing the enquiry. We are always pleased to help our numerous correspondents to the fullest extent, and we can trust them to sympathise in the overwhelming amount of writing which makes the new rules a necessity. Every communication must be accompanied by the writer's name and address, otherwise it will receive no attention.

### Queries.

(37) *Medical Examination.*—Information needed as to whether this is necessary for applicants for the Indian nursing service.—*E.F.*

(38) *Information Wanted.*—Where can I see Burdett's Hospital Annual?—*Welfare.*

(39) *Chemistry.*—I shall be glad of some advice regarding a boy of sixteen and a half just leaving school. He has a taste for chemistry. What steps should be taken to help him in this study?—*Waddon.*

(40) *The Nurse's Journal of the R.B.N.A.*—Is it true that this journal will not continue to be published?—*Sister.*

(41) *Maternity.*—I shall be glad to know if I can get free training in monthly nursing.—*L. M.*

(42) *Netley.*—To whom should I apply about entering the Army Nursing Service?—*Nurse.*

### Answers.

(37) *Medical Examination* (*E.F.*)—The form on which you will have to make your application supplies all the information asked for in your letter.

(38) *Information Wanted* (*Welfare*).—The book is published by the Scientific Press, 428, Strand.

(39) *Chemistry* (*Waddon*).—We should recommend you to write to the Secretary of the Pharmaceutical Society, Bloomsbury Square.

(40) *The Nurse's Journal.*—We are informed that a notice has been sent to members of the R.B.N.A. stating the number due in May will not be published. The whole question of its publication is under reconsideration. "The Nurse's Journal" fulfilled a useful purpose, and we hope it may be continued, as it ought to be easily possible to continue it, providing the finances of the R.B.N.A. are properly controlled, and economically administered.

(41) *Maternity* (*L.M.*).—No free training is given in monthly work.

(42) *Netley* (*Nurse*).—Write to the Director-General of the Army Medical Department, Victoria Street, Westminster.

### Our Examinations.

Some of the answers given to our last question are very good ones, but many have the fault of discursiveness. We should be very glad if our competitors would study to condense their replies, for without omitting any important details, at least half the words might be omitted in most instances without weakening the sense. We must also advise our friends to keep to the subject given. We are always sorry when a thoughtful correspondent disqualifies herself by writing down a quantity of practical hints, good enough in themselves, but absolutely unconnected with the examination question. Only the things relating to the one special case should be treated of each time. However, if any nurse feels that she would be glad to have an examination set on a special subject, she should write and make a suggestion to "Nursing," care of the Editor, and her wish would receive due consideration.

### Question for March.

Describe first aid and subsequent duty of a nurse towards a patient suffering from concussion of the brain.

### Prize Answer.

The unconscious state produced by a blow or fall on the head—or from a height on to the feet—is known as concussion, *i.e.*, a shaking of the brain; this may be slight or severe. In a slight case the patient is rendered unconscious only for a few minutes, but remains pallid, listless, and drowsy with pain in the head, and probably an attack of vomiting, until a period of rest and quiet, and above all sleep, have worked off the effects of the shock. If a nurse be called to attend a patient with the history of a recent fall or injury to the head, who is lying partially unconscious, only rousing when spoken to, with pallid face, cold clammy skin, shallow breathing, and weak irregular pulse, her first duty after sending for the doctor is to get the patient to bed in a darkened room, with warm blankets and hot bottles, and if possible ice or cold lotion to the head. If there be a wound she must shave off all the hair round and carefully cleanse with sterilised water or any antiseptic lotion which she can procure, then firmly apply a dressing. The utmost precautions ought to be taken in any scalp wound, especially when the bone is injured, to prevent the wound becoming septic; this might end in meningitis. Absolute quiet and low diet are essential in nursing cases of concussion; stimulants should be avoided; the bowels must be kept freely opened by means of aperients or simple enemas. If the patient gradually becomes quite insensible, with continued vomiting and loud stertorous slow respirations, the case is very serious. Concussion of the brain is often associated with fractured skull either of the vault or of the base, with compression, and the nurse can be of the greatest assistance to the surgeon by carefully noting any changes or further abnormal symptoms, such as bleeding from the mouth, nose, or ears, escape of watery fluid from the ears, contracted pupils, black eyes, twitchings or paralysis of the muscles of the face or of the limbs. In nursing a case convalescing from concussion of the brain the nurse must guard her patient from undue worry, over excitement, or fatigue.

Nurse G. A. Wilson.

### Question for April.

What would you do for a person who had a sunstroke in the cricket field or elsewhere if there were no doctor present?

Answers to be written *on one side of the paper only*, and sent in by April 24th, addressed "*Nursing*," Editor of The Hospital, 428, Strand, London.

### Wants and Workers.

Anyone sending a medical mission box abroad regularly, and who would like to enclose a copy of the *British Medical Journal* in it, is invited to write to *Sister, care of Manager*, The Hospital *Office*, 428, *Strand.*

Would any medical student or nurse like to share a subscription for the *British Medical Journal* for one year? It would be posted on regularly every Monday. Address *H. F. G., care of Manager*, 428, *Strand.*

Can anyone advise a healthy woman of sixty-four of any institution or agency through which she would be likely to get employment? She has hitherto worked as a nurse, but is told that her age is now a bar to her following this calling.—*M. D.*, 74, *Brondesbury Villas, Kilburn.*

### A Generous Bequest to Nurses.

On Monday, April 2nd, Dr. Samuel Johnson Moore died at his residence in Blythswood Square, Glasgow. A few days afterwards it was announced that after paying some bequests, the residue of his estate, amounting to not less than £45,000, is to be devoted to the foundation and maintenance of a Convalescent Home for nurses. The institution is to be known by the donor's name, and in admission a preference will be given to nurses who have been connected with the Glasgow Training Home for Nurses, Renfrew Street; but in an institution so well endowed this will in no degree exclude others. The intention is to provide a temporary home for nurses whose health has been either temporarily or permanently broken down in the exercise of their profession. The trustees are Sir John Burns of the Cunard Company, Mr. James Thompson, general manager of the Caledonian Railway Company and Dr. Hector Cameron, the well-known Glasgow surgeon, so there is no doubt that the fund will be managed with business prudence and professional consideration.

The life history of such a generous donor should be of interest to all nurses. Samuel Johnston Moore was practically a self-made man. He was an Irishman by birth, the son of a farmer in County Antrim. He was educated at Belfast Academy, and came to Scotland as a teacher in Paisley. Determined to advance, he began in 1859 to study medicine, although to do so he had to walk to and from Glasgow every day to attend the University classes, and pay for them by teaching at night in Paisley. In 1863 he graduated with first-class honours and was almost immediately appointed through the influence of Professor Allan Thompson, pathologist to the Glasgow Royal Infirmary. In the following year pathology was made compulsory for medical students, and Dr. Moore commenced an annual series of lectures on the subject. In 1869 he resigned his appointment on becoming an examiner in medicine. At the time of his death he was medical referee for the Caledonian and Glasgow and South-Western Railway Company. He was about sixty years of age.

In his family relations Dr. Moore was admirable. Throughout his own career, and even before it had become conspicuously successful, he was making efforts and sacrifices in order to help the younger members of his family, and his devotion to his mother was noteworthy. Until her death she shared his home, and his grave is by her side.

The disposition he has made of his property should win him the affectionate remembrance of all nurses, for, apart from its beneficent character, it is a testimonial to the value of their work from a member of that profession which best knows its value.

### Everybody's Opinion.

[Correspondence on all subjects is invited, but we cannot in any way be responsible for the opinions expressed by our correspondents. No communications can be entertained if the name and address of the correspondent is not given, or unless one side of the paper only be written on.]

### THE DAILY NURSE.

Miss Ethel McCaul writes: May I draw your attention to the fact that the Coventry Institution is not the only one in this neighbourhood which has started a plan of district nursing amongst the middle and upper classes. The Abbey Institute, Nuneaton, has recently organised a similar plan, which will undoubtedly prove a success and bring comfort to many. The class of patients who will chiefly benefit are those who either have not the accommodation or the means to engage a regular private nurse, and yet are not suitable for the ordinary district nurse. Maternity work will be an important branch of this daily nursing.

### For Reading to the Sick.

**Motto.**

Faith builds a bridge across the gulf of death.
—*Edward Young.*

**Verses.**

Faith alone is the master key
To the strait gate and narrow road ;
The others but skeleton pick-locks be,
And you never shall pick the locks of God.
—*Walter Smith.*

How can they live, how will they die,
How bear the cross of grief,
Who have not got the light of faith,
The courage of belief?—*Faber.*

Strong Son of God ! Immortal Love !
Whom we, that have not seen Thy face,
By Faith, and Faith alone, embrace,
Believing where we cannot prove. . . .
We have but Faith ; we cannot know,
For knowledge is of things we see
And yet we trust it comes from Thee,
A beam in darkness : let it grow !—*Tennyson.*

If thou could'st trust, poor soul,
In Him who rules the whole,
Thou would'st find peace and rest ;
Wisdom and sight are well, but trust is best.
—*A. Proctor.*

**Reading.**

All right-minded persons desire to be saved, to escape the doom of the impenitent sinner, and to come to that glorious Kingdom which God has prepared for them that unfeignedly love Him. How can this be done? If we feel our sins, and mourn over them, and know that unremoved they must bring us to destruction, what must we do to be saved? That was a question asked long ago by the Philippian gaoler, and the answer he received was, "Believe on the Lord Jesus Christ and thou shalt be saved." This is the way to escape the death in which unrepented sin will surely end, and to be accepted by God ; for, as it is declared in Holy Scripture, "there is none other name under Heaven given among men whereby we must be saved," but only the name of our Lord Jesus Christ. And Jesus Christ says of Himself, "I am the way, the truth, and the life, no man cometh to the Father but by Me." If, therefore, we would be saved, we must fix our faith on our blessed Redeemer, on Him who left His throne in Heaven, and took man's nature, and died for men's sins, died a bitter and cruel death upon the cross, and now, day by day, calls to every sinner who mourns over his past transgressions, "Come unto Me, all ye that labour, and are heavy laden, and I will give you rest." Yes! if we want rest from the burden of our sins, if we want salvation in this world and the next, we must not trust to any other person than Jesus Christ, or to any other way than faith in Him. If we feel that we have no faith, that we cannot see of what use He is to us, we must persevere in supplication till our eyes are opened. If we have a little faith and want more, we should say to God, "Lord, I believe, help Thou mine unbelief." Be not faithless but believing, and the spiritual treasures which will be opened will be of untold value, and our strength for good wonderfully great. . . . We have often read or heard read the life and death of our Lord Jesus Christ, as contained in the Holy Gospels; but as we read or heard, did it occur to us to think deeply, that all this life and all this death belongs to us? It is this personal interest in the facts we believe and profess which makes our religion and all that pertains to it of such priceless worth, and ourselves so strong in doing God's will. "Without faith it is impossible to please God"; but the faith which pleases God is that which follows on repentance. *Rev. R. Adams.*

### The Book World for Women and Nurses.

[We invite Correspondence, Criticism, Enquiries, and Notes on Books likely to interest Women and Nurses. Address, Editor, The Hospital (Nurses' Book World), 428, Strand, W.C.]

My China Sweetheart, and Other Poems. By Charles D. Steele. (The Ideal Publishing Union, Limited. London, 1894.)

Mr. Charles Steele has just collected together some of his many scattered verses which made their appearance in the *Merry-go-Round*, the *Quiver*, *Cassell's Monthly*, and The Hospital, the chaperonage of which magazines speaks for itself. These poems now form a neat little volume, which with a portrait of the author, and bound in white stamped paper can be obtained for the modest outlay of sixpence.

The Life of Princess Borghese. By Le Chevalier Zeloni. Translated by Lady Martin. (London: Burns and Oates, 1894.)

The Chevalier Zeloni has certainly performed his part of biographer with a degree of ardour almost unprecedented; one might almost say that this little volume was an "ecstasy" equally as a history. The Princess Borghese was a remarkable woman, whose good deeds and beautiful person claim our respect and our admiration alike. But it seems almost a question whether a volume, which deals in so exclusively personal a manner with her life would not have been better reserved for private circulation, and for the family archives. Would it, one asks, have pleased the gentle retiring disposition of Princess Gwendalin that the records of her private life, her letters and her diary should thus be laid bare to public view? As a translator, Lady Martin has done her work admirably, and the book is bound and printed in a manner which much redounds to the publishers' credit.

A Gauntlet. By Bjornstjerne Bjornson. Translated by Osman Edwards. (Longmans, Green, and Co., London.)

It is not to be expected that the English, nor indeed any public short of a Scandinavian one, will fully appreciate "A Gauntlet," nor indeed any of Bjornson's dramatic works. This the translator in his preface to "A Gauntlet" readily admits. On the stage of the Royalty Theatre in this year the play was little understood, its last chance of appealing to cultured and thoughtful minds being destroyed from the very facts that an adaptation was made with a view of rendering it more comprehensible to the ordinary playgoer. "A Gauntlet" stands a far better chance of meeting appreciation through the medium of Mr. Edwards' translation. "A Gauntlet" is a drama with a moral or purpose. Such works appeal to us better when quiet meditation and consideration is possible than in front of a stage, where we require our senses and imagination to be vividly and forcibly held. "A Gauntlet" deals with the subject of social purity. The scenes are cast in the home life of a middle-class household. The usual atmosphere of outward respectability prevails. The frail screen is drawn aside by a series of incidents, and the ideals of Svava, the heroine of the piece, are shattered. Through Svava's revolt against the evils revealed around her in her own home, and in those she holds most dear, Bjornson takes his opportunity of protesting against the dual standard of purity tacitly allotted to men and women. Bjornson wrote two versions of the play. In one Svava shows some signs of relenting and forgiving the lover who has so fallen short of her standard of right; in the other, the version before us, as translated by Mr. Edwards, Svava's adieu to her lover appears final indeed, for she flings her glove in his face. There is a comprehension of human nature, and an individuality of character very fascinating to follow through these pages of Bjornson. Very homely, simple words, incidents, and characters, but living beings speak to us through his pen, and they speak with the voice of nature, and that is the beginning and end of the best that art can attain.

### Books Received.

J. Hughes and Co.
Mrs. Lord's "Laundry Work for Scholars."

Seeley and Co.

"A Sketch of the Life and Character of Sarah Acland." Edited by Isambard Brunel.

Willing's "British and Irish Press Guide."

J. Curwen and Sons.
"Medburn Dumb-bell Exercises." Edited by F. W. Farrington.

Wright and Co., Bristol.
"The Medical Annual" for 1894.

**Periodicals and Pamphlets.**—Le Progrès Médical; Journal de Médicine; the Athenæum; Invention; the Rural World; the Building Societies Review; the Sun, Melbourne; London; the New York Medical Record; the Cornhill Magazine; Home Hospital for the Benefit of Gentlewomen; the First Principals of Voice Production, by Thomas Kelly.

### Where to Go.

Great Northern Central Hospital.—Bazaar at the hospital, Wednesday, April 18th.

Society for the Promotion of Female Welfare.—Sale of work at the Albert Hall, Thursday, April 19th.

The Ideal Club, Tottenham Court Road, Tuesday, April 17th, at three p.m., and at intervals till eight p.m. Addresses will be given to ladies on the principles of Reformed Clothing.

Lecturers' Club.—Women's Pioneer Lecturers, 4, Caroline Place, W.C. Mrs. Edward Senior will read a paper, at eight p.m., on Saturday, April 14th, on "French Peasant Life," with lantern illustrations. Admission to non-members by guest card 6d. each, to be obtained from the Hon. Secretary, Miss Hope Rea, 60, Belsize Park Gardens, N.W.

Exhibition of Modern Dutch Paintings. The Goupil Gallery, Regent Street, S.W.—Messrs. Bursod, Valadon, and Co. are exhibiting during April and May a collection of specimens of modern Dutch art, the merits of which collection is dependent on its quality rather than its quantity. Here we find four choice examples of Israels' work, of which, perhaps, No. 19, "Baby," is the most characteristic of the great master's brush. No. 31, "The Toy Boat," is another instance of the artist's consummately clever treatment of children, an art as strong as it is tender. Indeed, it is a question whether any living artist can approach Israels in his representation of childish form and expression. "Low Tide," No. 27, is a beautiful example of Dutch landscape by J. Maris, as is also "Rendez-vous des Vaches," No. 39, by W. Maris, his brother. As to technique and choice of subject-matter, a nation may well be proud of its artists who form the present collection at the Goupil Galleries.

Private View of the New English Art Club, Dudley Gallery.—The twelfth exhibition of modern pictures held by this club took place last Saturday. "Here," according to the words of one of its members, "he who has a story to tell let him tell it." In this particular instance, the walls of the Dudley Gallery are resplendent in the exemplification of this permission. There are many stories hanging here, in frames of quaint devising, but the reading of the same is reserved for the initiated alone—their language, alas, to many is foreign and unknown. For the public, more is the pity, are not awakened yet to an understanding of impressionistic work. The New English Art Club is leading the way in showing that there is an art even beyond the photography in colours, as presented yearly before our eyes in the various metropolitan picture galleries. Perhaps the most striking examples of works exhibited in the society's spring exhibition, are No. 39, "A Portrait," by Walter F. Cadly; No. 45, "A Dorsetshire Pastoral," by Bernhard Sickert; No. 66, a portrait of Mrs. Von Tunzelman, by Walter Sickert; and No. 84, "The End of an Afternoon, by Francis Bate. Mr. P. Wilson Steer contributes a clever study of a lady with auburn hair, against an equally warm background. In "Les Passades" Mr. Aubrey Beardsley is represented in a happily characteristic manner.

## Figures and Tables

**Figure f1:**